# Object Tracking Using Adaptive Covariance Descriptor and Clustering-Based Model Updating for Visual Surveillance

**DOI:** 10.3390/s140609380

**Published:** 2014-05-26

**Authors:** Lei Qin, Hichem Snoussi, Fahed Abdallah

**Affiliations:** 1 Institute Charles Delaunay, Université de Technologie de Troyes, 12 rue Marie Curie, CS 42060, 10004 TROYES CEDEX, France; E-Mail: hichem.snoussi@utt.fr; 2 Laboratory Heudiasyc, Université de Technologie de Compiègne, Rue Roger Couttolenc, CS 60319, 60203 COMPIEGNE CEDEX, France; E-Mail: fahed.abdallah@hds.utc.fr

**Keywords:** visual tracking, region descriptor, appearance model updating, clustering

## Abstract

We propose a novel approach for tracking an arbitrary object in video sequences for visual surveillance. The first contribution of this work is an automatic feature extraction method that is able to extract compact discriminative features from a feature pool before computing the region covariance descriptor. As the feature extraction method is adaptive to a specific object of interest, we refer to the region covariance descriptor computed using the extracted features as the adaptive covariance descriptor. The second contribution is to propose a weakly supervised method for updating the object appearance model during tracking. The method performs a mean-shift clustering procedure among the tracking result samples accumulated during a period of time and selects a group of reliable samples for updating the object appearance model. As such, the object appearance model is kept up-to-date and is prevented from contamination even in case of tracking mistakes. We conducted comparing experiments on real-world video sequences, which confirmed the effectiveness of the proposed approaches. The tracking system that integrates the adaptive covariance descriptor and the clustering-based model updating method accomplished stable object tracking on challenging video sequences.

## 1. Introduction

Visual tracking is an important and challenging task for building visual surveillance systems. In [1], a novel algorithm based on multiple sub-templates tracking is proposed for the terminal guidance application. In [2], both deterministic and probabilistic patch-based approaches are tested for observation detection for single object tracking.

In this work, we focus on the problem of tracking an arbitrary object with no prior knowledge other than an annotation in the first frame. Our goal is to develop an efficient and robust way to keep tracking the object throughout long-term video sequences in the presence of significant appearance variations and severe occlusions. Our idea for approaching robust visual tracking is of two folds: (1) build an object appearance model using descriptors or feature representations that have are discriminative and robust to extrinsic variations; (2) update the appearance model in a punctual and careful manner to keep the model adaptive to the intrinsic variations of the target.

The first contribution of this work is to study the generalization ability of the region covariance descriptor [3] from a machine learning perspective. We reveal that small eigenvalues in the eigenspectrum may degrade its generalization ability and therefore degrade the detection performance when using the descriptor directly. We then propose two remedies to tackle this problem: (1) by regularization to better conditionalize the covariance matrix; (2) by an automatic feature extraction method using the Principal Component Analysis (PCA) to extract compact relevant features before computing the region covariance descriptor. The second contribution is to propose a weakly supervised method for updating the object appearance model when tracking evolves. The clustering-based method performs a mean-shift clustering procedure among the accumulated tracking result samples and selects a group of reliable samples for updating the appearance model. As such, the method keeps the model adaptive to changes and prevents contaminating the model even in severely occluded scenes.

The rest of the paper is organized as follows. We review in Section 2 some previous work on feature descriptors and appearance model-based visual tracking. The region covariance descriptor is briefly reviewed in Section 3 with discussion. We present some variants of the covariance descriptor in Section 4. The advantages of the variants are empirically validated as well. We then build a tracking system using the newly proposed descriptor and a clustering-based model updating method in Section 5. The effectiveness of the proposed tracking system is evaluated by comparing experiments. The paper finishes in Section 6 with conclusions.

## 2. Previous Work

### 2.1. Feature Descriptors for Image Region Representation

Appearance feature description plays a crucial role in visual tracking, as the quality of the description directly affects the quality of the tracking performance. In general, the most desirable property of a feature description is to make the object easily distinguishable against non-targets in the feature space. From one pixel within a color image, the RGB color features can be naturally extracted. It is then not difficult to transform them into other color spaces or to gray levels. In addition, gradient and text features can also be extracted by considering the pixel within a local neighborhood.

In order to describe a region of pixels in a higher level, one popular way is to use a descriptor based on statistics, such as the histogram and the covariance matrix [3], which have been widely used in many computer vision applications to represent the pixel feature distribution.

The histogram descriptor is a nonparametric estimation of the distribution over pixel values in a region. It owns a simple form and shows good robustness against translation and rotation. In [4], a generalized histogram called spatiogram was proposed to capture not only the values of the pixels but also their spatial relationships as well. To calculate the histogram efficiently, [5,6] proposed intermediate representations to extract histograms called the integral histogram and the distributive histogram respectively. Although the histogram can accommodate any feature one at a time, the joint representation of several different features through histogram results in an exponential load as the number of features increases.

The region covariance descriptor [3], on the contrary, provides a natural way of fusing multiple features that might be correlated in a low dimensional covariance matrix. It can integrate the spatial, color and gradient information all in one matrix and disclose the correlation among them. It was shown in [3] that the covariance descriptor greatly outperforms histogram descriptor for object detection and texture classification.

Recently, sparse representation [7,8] has attracted increasing attentions in the computer vision community. It has been applied in face recognition [9], visual tracking [10–12], to name a few.

### 2.2. Appearance Models for Visual Tracking

The appearance representation of the target object implies a certain degree of constancy when transferring one frame to the next. Without any such constancy assumption, tracking cannot work. More precisely, it is assumed that the samples are generated from the same underlying probability distribution. Machine learning methods are then suitable and have been widely employed to fulfill visual tracking. The classifier learns to distinguish the target object based on its appearance model and a quantitative decision function.

When computing a classifier for object recognition, one faces two main philosophies, namely generative and discriminative models. The two categories can be described as follows. Given an input *x* and a label *y*, a generative classifier learns a model of the joint probability *p*(*x, y*) and classifies using *p*(*y*|*x*), which is obtained by using the Bayes rule. In contrast, a discriminative classifier models the posterior *p*(*y*|*x*) directly from the data or learns a map from the input *x* to the label *y*: *y* = *f*(*x*).

For visual tracking, the background is “the rest of the world” except the target object, which is too wild to model its class conditional distribution. Therefore, most generative models in the literature only model the target object and totally ignore the background. In this sense, generative trackers represent the appearance of an object by learning a model that provides sufficient reconstruction ability. Tracking is expressed as finding the most similar object appearance to the model. As they model only the target object, techniques employed are generally unsupervised, such as Principal Component Analysis (PCA) [13], Independent Component Analysis (ICA) [14], Mixture Models [15], Expectation Maximization (EM) [16] and Sparse Representation [8]. To handle the variability of a target, the object model is often updated online to adapt to appearance variations. Some representative generative trackers can be found in [17–22].

For trackers that adopt discriminative models, a classifier is trained directly from training samples to find a decision boundary that best distinguishes the object from the background. This type of methods is also known as “tracking-by-detection”, where a target object, identified by the user in the first frame, is described by a set of features. A binary classifier separates the target from the background in successive frames. Classification tools employed by discriminative methods are typically supervised techniques, such as Linear Discriminant Analysis (LDA) [23], Support Vector Machine (SVM) [24], Boosting [25], Random Forests [26], as well as their variants. When properly trained, discriminative methods can demonstrate robustness to avoid distracters in the background, in contrast to their generative counterparts. Some representative discriminative trackers can be found in [27–32].

For detailed surveys of appearance models for visual tracking, we refer the readers to [33–36].

## 3. Review of the Region Covariance Descriptor

We shall make a brief review of the region covariance descriptor and then discuss its properties and problems.

### 3.1. Region Covariance Descriptor

The region covariance descriptor was firstly proposed by Tuzel *et al.* in [3]. The idea is to represent a feature distribution using its sample covariance matrix.

Let *I* be a *W* × *H* one-dimensional intensity or three-dimensional color image, and *F* be the *W*×*H*×*d* dimensional feature image extracted from *I*

(1) F(x,y)=Ψ(I,x,y)

where Ψ is a function extracting image features such as intensity, color, gradients, and filter responses, *etc.* For a given rectangular region *R* ∈ *I*, denote {*f_i_*}*_i_*_=1,…,_*_N_* as the *d*-dimensional feature points obtained by Ψ within *R*. The region *R* is then represented by a *d* × *d* covariance matrix:

(2) $$C_{R}=\frac{1}{N-1}\sum_{i=1}^{N}(f_{i}-\mu ){\left(f_{i}-\mu \right)}^{⊤}$$

where *μ* is the mean vector of {*f_i_*}*_i_*_=1…_*_N_*.

For fast calculation of covariance matrices, [3] also provided an intermediate representation called integral image. With this representation, covariance descriptor of any rectangular region can be computed within constant time [3].

Covariance matrices do not lie on the Euclidean space. Therefore, an arithmetic subtraction of two matrices would not measure the distance of the corresponding regions. In fact, nonsingular covariance matrices are Symmetric Positive Definite (SPD) and lie on a connected Riemannian manifold. Accordingly, Riemannian metrics should be used for computing distance and mean of covariance matrices. There are two Riemannian metrics proposed in the literature. One is the affine-invariant Riemannian metric presented in [37,38]. The other is the bi-invariant log-Euclidean metric introduced in [39].

### 3.2. Discussion

Most applications that employ the covariance descriptor compute the descriptor using a fixed set of features, which is often determined a priori. For instance, in [3], each pixel is converted to a nine-dimensional feature vector for object detection:

(3) $$f(x,y)=[xyR(x,y)G(x,y)B(x,y)\left|I_{x}(x,y)\right|\left|I_{y}(x,y)\right|\left|I_{xx}(x,y)\right|\left|I_{yy}(x,y)\right|]$$

where *R, G, B* are the three color channels in the RGB color space, *I* denotes the pixel intensity and *I_x_, I_xx_, I_y_, I_yy_* are the first- and second-order image derivatives of *I* with respect to the Cartesian coordinates *x* and *y* respectively. This feature set remains unchanged in [3] for all kinds of objects, without considering the characteristics of each object.

Actually, color can be interpreted and modeled in different ways. With the availability of a variety of color spaces, e.g., RGB, HSV, YCrCb, YUV, CIE Lab, CIE Luv, *etc.*, the inevitable question is how to select proper color models that can produce good performance for detecting a particular object. Likewise, the gradient features, which encode the shape information of the region context, can also have a variety of choices. Indeed, they can be computed using different combinations of orders, and further with their corresponding magnitudes and orientations. Consequently, how to choose the feature set to be fused in the covariance descriptor for detection is of great importance.

A number of works [40–43] have empirically studied the performances of the covariance descriptor using different feature sets. The reported results showed that significantly different performances were achieved when using different features. This further shows the importance of feature selection or extraction for the covariance descriptor. Alahi *et al.* [40,41] compared different feature sets for detection and tracking objects across non-calibrated camera networks and claimed that increasing the number of features may increase the performance of covariance descriptor. In addition, Alahi *et al.* suggested that shape information is crucial for inter-category object detection [40]. For instance, gradient features perform well in pedestrian detection applications because the shape of a human is a relevant cue to distinguish it from other objects, whereas color features perform best in intra-category classification cases such as object re-identification or recognition. In [42], Cortez-Cargill *et al.* constructed covariance descriptors with nine sets of features based on various color spaces. They obtained a best feature set that embeds many color channels from a variety of color spaces and reaches a performance of 99% for face detection. However, the feature vector they got turned out to have 20 dimensions, which makes the construction and similarity measurement of the covariance matrices rather time-consuming.

In brief, two points can be drawn. First, different feature combinations generally produce different detection performances. Second, previous works generally reported better results using more features. Subsequently, two questions naturally arise. First, how to select proper feature set for detecting a specific object to ensure good performance in terms of detection accuracy? Second, is it always true that fusing more features produces better detection performance? If so, are there alternatives that use compact feature set while ensuring good performance? If not, what is the condition when more features do not yield better performance? We will try to answer these questions in the next section by analyzing the generalization ability of the region covariance descriptor from a machine learning perspective.

## 4. Variants of the Region Covariance Descriptor

### 4.1. A Machine Learning Perspective of the Region Covariance Descriptor for Object Detection

The essence of image region matching is to measure the similarity between the object template and a candidate image patch. Region descriptors using statistics of the pixel set are designed to represent feature distribution of the pixels inside an image region. As such, the similarity between feature distributions is reduced to comparing the distance of the corresponding region descriptors. Object detection using region descriptors takes the underlying assumption that descriptors computed from image regions that contains the same object have smaller distances than those computed from non-targets. This is indeed a machine learning process. With a training set of the object template, one seeks to compute statistics to characterize the feature distribution of the object. Two typical statistics that represents feature distribution based on training samples are the histogram and the covariance matrix.

For object detection, one has a training set, *i.e.*, the pixel set of the object template. Each pixel is represented by a vector of features that are extracted from the image. As such, statistical models can be learned from this training set in order to represent the object to be detected. In this sense, the region covariance descriptor estimates the covariance of the feature distribution using the training sample set. It then estimates the variances of the features in the diagonal entries of the matrix and the covariances between pairs of features in off-diagonal entries to represent the second order statistics of the pixel feature distribution. Testing a candidate sample is conducted by measuring the distance between corresponding region descriptors. For the region covariance descriptor, this can be done either using the affine-invariant metric or the log-Euclidean metric. It is worth noting that both of the Riemannian metrics compute logarithm of eigenvalues in order to transform a point from the SPD Riemannian manifold into a local Euclidean space.

The detection performance of the descriptor depends mainly on the generalization ability of the model, which can be analyzed by means of the bias and variance decomposition. First, the eigenvalues are estimated from limited training samples. It is well known that the estimates based on Equation (2) produces biased estimates of the eigenvalues; the largest ones are largely biased and the smallest ones are biased towards values that are too low. This bias is most pronounced when the population eigenvalues tend towards equality and is correspondingly less severe when their values are highly disparate. In all cases, this phenomenon becomes more pronounced as the sample size decreases [44–46]. Second, if there are very small eigenvalues in the sample covariance matrix, logarithm of these tiny eigenvalues will incur large disturbance that can dramatically degrade the generalization ability.

Therefore, it is important to analyze the eigenspectrum of the sample covariance matrix of the object template. If there are some very small eigenvalues, reduction of incurred variance is necessary. To this end, we propose below two remedies for poorly-conditioned covariance matrices, one by regularization and the other by dimension reduction.

### 4.2. Regularized Covariance Descriptor

To cure the large variance problem caused by tiny eigenvalues, our first solution is to use regularization techniques, which have been highly successful in the solution of ill- and poorly-posed inverse problems. Specifically, we regularize the estimated covariance matrix by adding a scaled identity matrix to it, *i.e.*,

(4) $$C_{R}=C_{R}+\eta E$$

where *E* is the identity matrix that has the same size as *C_R_*. With sufficiently large *η*, this regularization can effectively cure the poorly-conditioned sample covariance matrix. We name the resulting region covariance descriptor after regularization as the “regularized covariance descriptor”.

Regularization reduces the variance associated with the sample-based estimate at the expense of potentially increased bias. Hence, the choice of the value of *η* is of importance. Generally, over-regularization using large *η* will introduce large bias, whereas under-regularization will not effectively cure the large variance problem. To determine a proper value for *η*, one needs to take into account several factors, e.g., the slope of the log function, the range of the feature channels, among others.

### 4.3. Adaptive Covariance Descriptor

Another way to reduce variance caused by tiny eigenvalues is to use PCA projection to remove those unreliable dimensions while preserving dominant information in the principal components.

Specifically, we first extract raw features from the image patch to form a set of *n d*-vectors (*n* indicates the number of pixels in this region and *d* is the dimension of features); based on this point set, we not only construct the original covariance matrix but also learn a PCA projection. The *d*-dimensional dataset is then projected to a subspace by the learned PCA projection yielding a compact *k*-dimensional point set. Finally, the adaptive covariance matrix descriptor is constructed using the projected point set.

For a candidate image patch to be compared with the template, the descriptor computation is similar except that it employs the PCA projection pre-learned from the template point set. In this way, the feature extraction is adaptive to a specific target. We name the region covariance descriptor computed in this fashion as the “adaptive covariance descriptor”.

#### 4.3.1. Computation of the Adaptive Covariance Descriptor

In the training stage, based on a *d*-dimensional feature pool and the point set from the object template image, the PCA projection matrix is learned by keeping the *k* (1 ≤ *k* ≤ *d*) top eigenvectors of the sample covariance matrix according the significance of their corresponding eigenvalues. The mean vector of the training samples is preserved as well.

When generating the adaptive covariance descriptor, each point represented by *f*(*x, y*) is firstly subtracted by the mean of the training samples. Then, it is projected to the subspace spanned by the *k* retained eigenvectors, yielding a compact *k*-dimensional feature vector *p*(*x, y*). Finally, the adaptive covariance descriptor of an image region is computed using the sample covariance of the extracted feature vector *p*(*x, y*)

(5) $$C_{a,R}=\frac{1}{N-1}\sum_{i}^{N}p_{i}\times p_{i}^{⊤}$$

We summarize the procedure in Algorithm 1.


**Algorithm 1** Procedure for computing the adaptive covariance descriptor.
**Training Stage:****Input:** target template image from the initial frame**Output: ***μ*(*f*): the mean feature vector; *V_k_*: the projection matrix.1:Form the pixels that are inside the template image.2:Extract feature vector *f_i_* ∈ ℜ*^d^*^×1^ for each pixel *i*.3:Compute the mean vector: *μ*(*f*) ← *mean*(*f_i_*).4:Compute the covariance matrix: *C_R_* ← *cov*(*f_i_*).5:Do eigenvalue decomposition for *C_R_*: *C_R_* = *V*Λ*V*^T^.6:Keep the *k*(0 ≤ *k* ≤ *d*) eigenvectors *υ*_*i*=__1⋯_*_k_* in *V* that correspond to the *k* most significant eigenvalues: *V_k_* ← [*υ*_1_ ⋯ *υ_k_*]**Generating Descriptors**:**Input:** any image region *R, μ*(*f*), *V_k_***Output: ***C_a,R_*: the adaptive covariance descriptor of the region *R*1:Form the pixels that are inside the region *R*.2:Extract feature vectors *f_i_* for each pixel *i*.3:Perform the PCA projection on each *f_i_* and obtain a compact score vector *p_i_* ∈ ℜ^*k*×^^1^:
$p_{i}\leftarrow V_{k}^{⊤}(f_{i}-\mu (f))$.4:Compute the adaptive covariance descriptor *C_a,R_*∈ ℜ*^k^*^×^*^k^* using the sample covariance matrix of *p_i_*: *C_a,R_* ← *cov*(*p_i_*).


Note that since the adaptive covariance descriptor is still a covariance matrix, the integral image [3] can be naturally inherited for fast covariance matrices computation.

It is pointed out in [3] that for the conventional covariance descriptor, given a region *R*, its covariance *C_R_* does not have any information regarding the ordering and the number of points, which implies a certain scale and rotation invariance over the regions in different images. However, if the matrix fuses the information regarding the orientation of the points, such as the norm of gradient with respect to *x* and *y*, the covariance descriptor is no longer rotationally invariant. The same argument is also correct for scale and illumination. The features fused in the adaptive covariance descriptor are linear combinations of the raw features. Therefore, its invariance property is the same as the conventional descriptor. That is, if the raw features are scale, rotation or illumination invariant, then the adaptive covariance descriptor is also scale, rotation and illumination invariant. Otherwise, invariance does not hold.

#### 4.3.2. Relation to the Conventional Covariance Descriptor

We explore in this section the relationship between the conventional covariance descriptor and the proposed adaptive covariance descriptor in order to elucidate the superior representation ability of the proposed descriptor.

Let *C_r_* denote the conventional covariance matrix descriptor computed for the target template image (the reference) and *C_c_* denote that of an arbitrary candidate image region to be matched with *C_r_*. The distance between *C_r_* and *C_c_* under the log-Euclidean metric [39] is:

(6) $$d(C_{r},C_{c})=\left\|\log(C_{r})-\log(C_{c})\right\|$$

After the PCA projection, the adaptive covariance descriptor for the target template becomes

(7) $$C_{a,r}=V_{k}^{⊤}C_{r}V_{k}$$

Similarly, the adaptive covariance descriptor for the candidate image becomes

(8) $$C_{a,c}=V_{k}^{⊤}C_{c}V_{k}$$

The distance between *C_a,r_* and *C_a,c_* is thus:

(9) $$\begin{array}{ll} d(C_{a,r},C_{a,c}) & =\left\|\log(V_{k}^{⊤}C_{r}V_{k})-\log(V_{k}^{⊤}C_{c}V_{k})\right\|\\ & =\left\|V_{k}^{⊤}\cdot (\log(C_{r})-\log(C_{c}))\cdot V_{k}\right\| \end{array}$$

It is interesting to find that if all the eigenvectors are kept, *i.e., k* = *d*, the new distance in Equation (9) is equal to the original distance (6). This equality indicates that rotation of coordinate systems using PCA projection does not affect distances between covariance matrices. Nevertheless, this rotation by PCA projection is of interest, because it provides a most “suitable” coordinate system for analyzing the fused features from the perspective of the target template image.

In other cases where only a few principal components are kept, the equality of Equations (6) and (9) no longer holds. The dimension reduction of PCA removes unreliable dimensions and thus makes the adaptive covariance descriptor different from the conventional one. From a machine learning perspective, the PCA projection during the computation of the adaptive covariance descriptor preserves dominant information and removes noise. Removing the unreliable dimensions can alleviate the overfitting problem and hence improve generalization.

Compared with the conventional descriptor, another advantage of the adaptive descriptor is that it is more compact. As such, it can vastly accelerate the subsequent operations, e.g., distance measurement and appearance model updating. We acknowledge that the adaptive covariance descriptor may impose additional computational burden for training the PCA and projecting the raw feature vectors into principal components. However, this additional computational effort for obtaining a compact representation is well worth it. Firstly, training is an offline process, which is performed only once at the first frame. The computation during the training stage for learning the PCA is thus negligible. Secondly, the benefit of the compact representation in subsequent processing may outweigh the cost of the PCA projection. In the practice of object detection, hundreds of thousands of candidate descriptors need to be computed and compared for a test image. As such, employing the compact representation of the adaptive descriptor can result in significant efficiency gain.

In brief, the adaptive covariance descriptor represents an object using a covariance matrix that fuses a few relevant features formed by the principal components of the raw feature distribution. Compared with the conventional descriptor, operations on the adaptive descriptor are generally faster. Furthermore, if the conventional descriptor has very small eigenvalues, the adaptive descriptor should have better generalization ability than the conventional one.

### 4.4. ℓ_1_ Norm for Distance Measure

Since the logarithm domain of the SPD matrices manifold is in Euclidean space [39], one may consider using ℓ_1_ norm instead of the ℓ_2_ norm to measure the distance of two matrices in the logarithm domain. The intuition is that the ℓ_1_ is generally more robust to outlier than the ℓ_2_ norm [47]. We can thus expect the modified distance metrics using ℓ_1_ norm to yield better detection performances.

Specifically, the two Riemannian metrics are modified as follows. For the affine-invariant metric, the distance between two covariance matrices are modified as

(10) $$\rho (C_{1},C_{2})=\sum_{i=1}^{d}\left|(\lambda_{i}(C_{1},C_{2})\right|$$

where | · | is the abstract value function. Likewise, the log-Euclidean metric can be modified using ℓ_1_ norm as

(11) $$d(C_{1},C_{2})={\left\|\log(C_{1})-\log(C_{2})\right\|}_{\ell_{1}}$$

where ‖ · ‖ _ℓ1_ is the ℓ_1_ norm by taking the matrix log(*C*) as vector.

### 4.5. Empirical Evaluation

In order to validate the effectiveness of the claimed improvements to the conventional region covariance descriptor, we empirically assessed the performances of the regularized descriptor and the adaptive descriptor in comparison with that of the conventional descriptor by repeatedly detecting objects in real-world video sequences.

**Figure 1.**
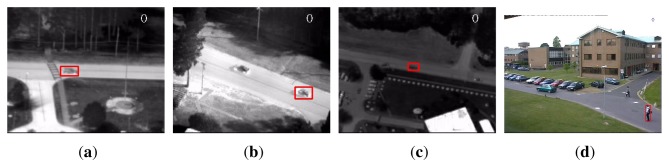
Initial frame of each sequence with target marked in rectangle. (**a**) PkTest01; (**b**) PkTest02; (**c**) PkTest03; (**d**) PETS.

As a benchmark, three publicly available sequences, namely “PkTest01”, “PkTest02” and “PkTest03”, from the VIVID airborne sensor dataset [48,49] were used for evaluation. The three sequences from VIVID dataset are thermal infrared data of vehicles captured by moving cameras in airport circumstances. These sequences are selected because there is strong correlation between the color channels. Therefore, if many color features are used, there will be some very small eigenvalues in the covariance matrix. In addition, there are similar vehicles in the scene, making the detection challenging. As the sequences are very long, we used only the first 100 frames of each sequence. In addition, a public color sequence from the PETS dataset [50] is used as well. The sequence is captured from a static camera in a campus circumstance, where we seek to detect a walking pedestrian. There are some other pedestrians in the scene. Similar to the VIVID sequences, we used only 100 frames (from Frame #1412 to Frame #1511) of the sequence. Figure 1 displays the target objects, marked in rectangles in the first frames.

#### 4.5.1. Settings

Using the annotated template image in the first frame, we first computed the three descriptors, *i.e.*, the conventional descriptor, the regularized descriptor and the adaptive descriptor of the object respectively. As in [3], an object was represented by five covariance matrices of the image features computed from five sub-regions (the whole region, the left half part, the right half part, the top half part and the bottom half part) of the object template image. Then, we used each of the descriptors to detect the object in the rest of the sequence and evaluated its performance.

For all the sequences, we used a feature set *f*(*x, y*) defined as

(12) $$f(x,y)={\left[R(x,y)G(x,y)B(x,y)H(x,y)L(x,y)S(x,y)a(x,y)b(x,y)u(x,y)v(x,y)\frac{\partial I(x,y)}{\partial x}\frac{\partial I(x,y)}{\partial y}\frac{\partial^{2}I(x,y)}{\partial x^{2}}\frac{\partial^{2}I(x,y)}{\partial y^{2}}\frac{\partial^{2}I(x,y)}{\partial x\partial y}\frac{\partial^{3}I(x,y)}{\partial x^{2}\partial y}\frac{\partial^{3}I(x,y)}{\partial x\partial y^{2}}\frac{\partial^{4}I(x,y)}{\partial x^{2}\partial y^{2}}\right]}^{⊤}$$

where *H*(*x, y*), *L*(*x, y*) and *S*(*x, y*) are the feature channels from the HLS color space. Similarly, *a*(*x, y*), *b*(*x, y*) and *u*((*x, y*), *υ*(*x, y*) are from the CIE Lab and CIE Luv color spaces respectively. The *L* channels in Lab and Luv colors spaces are not used because they are highly correlated with each other and also with the *L* channel in the HLS space. Note that all the color channels need to be adjusted to fall into the range of 0–255. The derivatives of the intensity image are computed as they are using the Sobel operator with 3 × 3 or 5 × 5 kernels (According to the summarized order of the partial derivatives, *i.e.*, if the summarized order is less than 3, we used the 3×3 kernel; otherwise the 5×5 kernel was used.).

The conventional descriptor used this set directly. The regularized descriptor used the same feature set *f*(*x, y*). The parameter *η* was set to 0.5. For computing the adaptive descriptor, the number of retained principal components after PCA projection was automatically determined. Those dimensions with eigenvalues less than 0.01 were removed.

The search method for locating the object is also similar to that in [3]. Initially, we computed only the descriptor of the whole region. We search the target image for a region having similar covariance matrix. The search was performed by sliding-window from left to right and from top to bottom in the whole image frame. The window size was fixed as that of the template image with no scale change. The search window jumped horizontally 10% of the width or vertically 10% of the height of the object between two search locations. After this first phase, we kept the best matching 1000 locations. At the second phase we repeated the search in the 1000 detected locations using all five covariance matrices. The dissimilarity of the object and a candidate region was computed by summarizing the distances of all five pairs of covariance matrices. Finally, the region with the smallest distance was selected as the matching region. We used the log-Euclidean metric to measure the distances between covariance matrices. Our implementation is in C++ and is based on the OpenCV library.

Two quantities were measured to evaluate the performance of the descriptors. One is the detection rate, which is defined as the ratio of the number of frames where object location is accurately estimated to the total number of frames for detection. The detection result is considered to be accurate if the center position of the best match is within the 9 × 9 pixel neighborhood of that of the ground truth. The other metric is the average processing time per frame, employed to evaluate the computational efficiency.

#### 4.5.2. Results

Table 1 summarizes the detection rates and the average processing time per frame using the conventional covariance descriptor (denoted as “Cov”), the adaptive covariance descriptor (denoted as “AdpCov”), and the regularized covariance descriptor (denoted as “RegCov”).

For comparing the ℓ_1_ norm and the ℓ_2_ norm, performances using each norm are presented respectively. As such, in Table 1, “Cov1” denotes the utilization of conventional covariance descriptor using the log-Euclidean metric with ℓ_1_ norm, *i.e.*, Equation (11), whereas “Cov2” denotes the utilization of the log-Euclidean metric with the ℓ_2_ norm. Other notations are similar. Note that the average processing time is the total average of both ℓ_1_ and ℓ_2_ norms.

**Figure 2.**
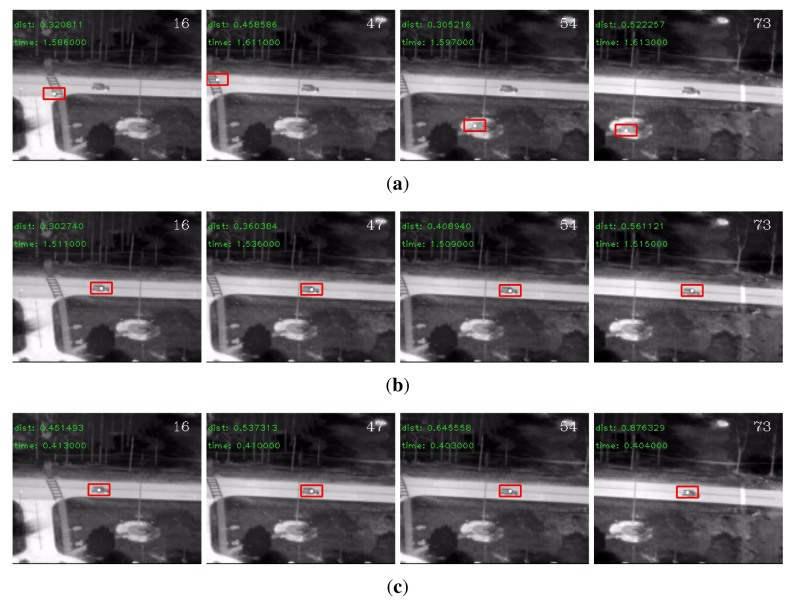
Some detection results for the “PkTest01” sequence (**a**) using the conventional covariance descriptor, (**b**) using the regularized covariance descriptor and (**c**) using the adaptive covariance descriptor. The initial frame with the target object marked in rectangle is shown in Figure 1a.

**Figure 3.**
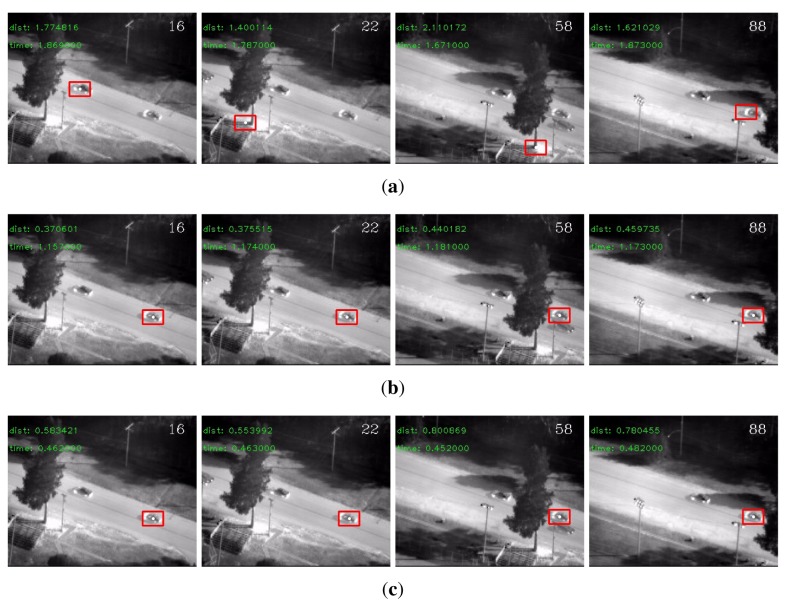
Some detection results for the “PkTest02” sequence (**a**) using the conventional covariance descriptor, (**b**) using the regularized covariance descriptor and (**c**) using the adaptive covariance descriptor. The initial frame with the target marked in rectangle is shown in Figure 1b.

On the VIVID sequences, there were a number of very small eigenvalues in the eigenspectrum of the conventional descriptor. The detection rates clearly showed that these small eigenvalues degraded the performance of the conventional descriptor. The regularized descriptor and the adaptive descriptor both handled this problem effectively and boosted the detection rates. Besides, the ℓ_1_ norm generally outperformed the ℓ_2_ norm. In terms of efficiency, benefited from the reduced dimensionality, the adaptive descriptor was significantly faster than the other two descriptors. Therefore, if efficiency is a major concern, the adaptive covariance is indeed a good choice. We display some detection results by each descriptor for the “PkTest01” sequence in Figure 2 and those for the “PkTest02” sequence in Figure 3 respectively. Since the ℓ_1_ norm generally performed better than the ℓ_2_ norm, the presented results are those detected by the ℓ_1_ norm.

**Figure 4.**
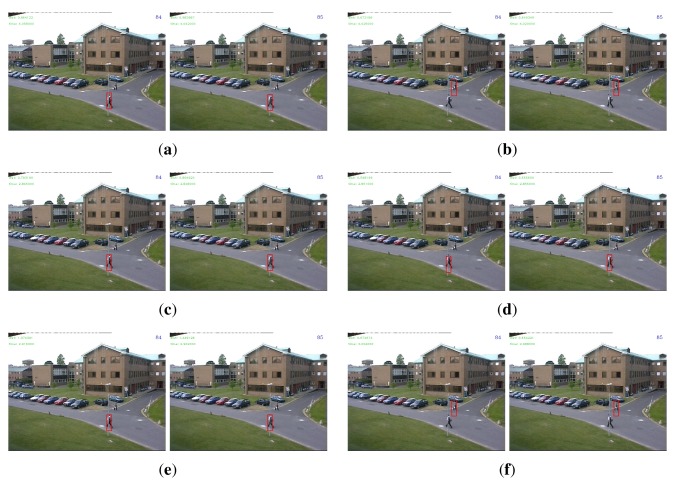
Detection results in the 84th and the 85th frames of the “PETS” sequence. See text for details. The initial frame with the target marked in rectangle is shown in Figure 1d. (**a**) Cov1; (**b**) Cov2; (**c**) RegCov1; (**d**) RegCov2; (**e**) AdpCov1; (**f**) AdpCov2.

For the PETS sequence, there were no very small eigenvalues in the eigenspectrum of the conventional covariance descriptor of the target object. Indeed, all eigenvalues were greater than 0.1. Therefore, no dimensions were removed for the adaptive covariance descriptor. In terms of accuracy, we see that all descriptors performed quite well with detection rates from 98% to 100%. The slight performance deterioration is due to a partial occlusion in the 84*^th^* and 85*^th^* frames (Frame #1496 and Frame #1497 in the original sequence). The ℓ_1_ norm successfully overcame this problem for all three descriptors, while the ℓ_2_ norm drifted to another pedestrian in the scene except for the regularized descriptor. This phenomenon is displayed in Figure 4, which further demonstrated that the ℓ_1_ norm is more robust to outliers than the ℓ_2_ norm. In terms of efficiency, as dimensionality was not reduced, the adaptive descriptor was slightly slower than the regularized descriptor due to the extra computational burden of the PCA projection. A mysterious observation is that for this sequence, the conventional descriptor was much slower than the other two descriptors. Similar phenomenon can also be noticed for the three VIVID sequences: the regularized descriptor was generally faster than the conventional descriptor. A plausible reason is that when the covariance matrices are poorly-conditioned (even though the descriptor of the target object is not poorly-conditioned, there may be poorly-conditioned descriptors among the numerous candidate regions), the implementation routine that performs the matrix logarithm operation conducts some extra computation to improve stability and thus makes the conventional descriptor less efficient.

#### 4.5.3. Discussion

The above experiments show that small eigenvalues indeed degrade the generalization ability of the conventional covariance descriptor. In general, a fixed feature set cannot always work well in all circumstances. The analysis of the eigenspectrum of the conventional covariance descriptor is thus important for detecting the small eigenvalue problem. Once detected, the proposed two variants are both effective to cure this problem.

Another perspective of the adaptive covariance descriptor is that it uses PCA to extract relevant compact features that are adaptive to a specific object. Irrelevant features are discarded. On the contrary, the regularized descriptor tries to alleviate the adverse effect of the irrelevant features, with the relevant features almost unaffected.

It is also interesting to draw an analogy between the two variants of the covariance descriptor and those of the linear least squares regression. For regression from *X* to *Y*, the ordinary least squares (OLS) solution is (*X*^⊤^*X*)^−^^1^*X*^⊤^*Y*. When *X*^⊤^ *X* is ill- or poorly-conditioned, one can either use the ridge regression or use the principal component regression (PCR) to handle this problem. The rigid regression is analogous to the regularized descriptor here and the PCR is analogous to the adaptive descriptor. This correspondence can be established because both the function *y* = 1/*x* and the function *y* = log (*x*) have sharp slopes when *x* is very close to zero, which makes the results unstable.

## 5. Object Tracking

In this section, we shall build a tracking system that integrates the newly proposed adaptive covariance descriptor and a new model updating method. First, the object appearance model using multiple patches is presented in Section 5.1. Second, target localization using the appearance model and similarity measurement is addressed in Section 5.2. Most importantly, for updating the model during the tracking, we propose in Section 5.3 a weakly supervised updating method, which is based on clustering analysis using the mean-shift procedure.

### 5.1. Object Appearance Model

**Figure 5.**
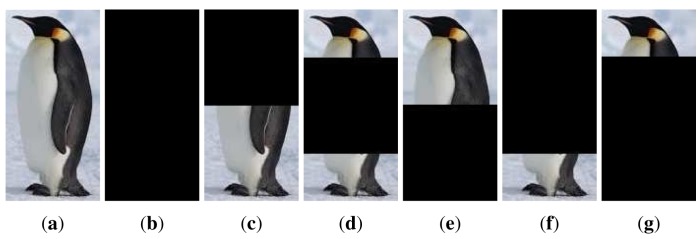
Illustration of the multiple-patches object representation. An object on the left is represented by six adaptive covariance descriptors computed from corresponding sub-regions on the right. Note that if the width of the object is greater than its height, a similar division is performed in the horizontal direction.

To increase robustness, we use multiple patches of an image region, each of which is described by an adaptive covariance descriptor. A simple heuristic is employed to divide the object into six parts. If the width of the object is smaller than the height, the object is divided in the vertical direction. Otherwise, it is divided in the horizontal direction. This multi-part representation mechanism is illustrated in Figure 5, where an object on the left is vertically divided into parts on the right because its height is greater than its width.

As such, a region is represented by six adaptive covariance matrices computed from its six sub-region patches. Each patch is then represented by an adaptive covariance descriptor, denoted as ${\left\{C_{a}^{i}\right\}}_{1\leq i\leq 6}$. For instance, when the height is greater than the width, $C_{a}^{1}$ is computed from the entire region as in Figure 5b; $C_{a}^{2}$ from the top half as in Figure 5c; $C_{a}^{3}$ from the middle half as in Figure 5d; $C_{a}^{4}$ from the bottom half as in Figure 5e; $C_{a}^{5}$ from the top 3/4 part as in Figure 5f and $C_{a}^{6}$ from the bottom 3/4 part as in Figure 5g. For objects that have width greater than height, correspondence can be naturally established.

Although computed in a subspace, the adaptive covariance descriptor is indeed a sample covariance matrix, which lies on a Riemannian manifold. Using the log-Euclidean transformation [39,51,52], we first transform the adaptive descriptors of the six patches $C_{a}^{i}(i=1\cdots 6)$ to Euclidean space as log $C_{a}^{i}(i=1\cdots 6)$, then unfold each matrix and concatenate them to take a vector form. Note that since the transformed matrices log *C_a_* are still symmetric, only upper triangular matrices are used. For instance, if 10 out of 15 dimensions are retained when computing the adaptive covariance descriptor, the dimension of the final vector representation of a region is 10 × (10 + 1)/2 × 6 = 330, whereas the conventional covariance descriptor using 15 features would generate a vector of size 15 × (15 + 1)/2 × 6 = 720.

Using the adaptive covariance descriptor and the transformations above, we obtain a vector-form feature representation of the target object, denoted as *M_r_*, as the object appearance model. The appearance model learned from the initial target template image, denoted as $M_{r}^{0}$, is preserved throughout the tracking process for later participating the updating of *M_r_*.

### 5.2. Target Localization

To track the target in consecutive frames, we use a uniform sliding-window search around the target's previous position. That is, our motion model is such that the location of the tracker at time *t* is equally likely to appear within a rectangle window around the tracker location at time (*t* − 1). Let ℓ*(*t* − 1) denote the tracker location at time (*t* − 1), $x\left(\ell_{t-1}^{*}\right)$ be the *x* coordinate of ℓ*(*t* − 1) and $y\left(\ell_{t-1}^{*}\right)$ be the *y* coordinate of ℓ*(*t* − 1). The motion model is formally defined as

(13) $$p(\ell_{t}|\ell_{t-1}^{*})\propto \{\begin{array}{ll} 1 & \text{if}\left\|x(\ell_{t})-x(\ell_{t-1}^{*})\right\|<s_{1}\text{and}\left\|y(\ell_{t})-y(\ell_{t-1}^{*})\right\|<s_{2}\\ 0 & \text{otherwise} \end{array}$$

where *s*_1_ and *s*_2_ are predefined constants that constrain the boundaries of the searching area.

At time *t*, when a new image frame is captured, a number of candidate regions are generated according to the motion model (13). Similar to the target object, each candidate region is represented using six adaptive covariance descriptors and then transformed to a vector-form feature representation. We denote the feature representation of the *i*t̂h candidate region as $M_{c,t}^{i}$. The distance between $M_{c,t}^{i}$ and the current target appearance model *M_r_* is measured by

(14) $$d(M_{r},M_{c,t}^{i})={\left\|M_{r}-M_{c,t}^{i}\right\|}_{\ell_{1}}$$

where ‖ · ‖_ℓ_1__ is the ℓ_1_ vector norm.

The best match is the candidate region whose feature representation $M_{c,t}^{*}$has the smallest distance to *M_r_, i.e.*,

(15) $$M_{c,t}^{*}=\arg\underset{i}{\min}d(M_{r},M_{c,t}^{i})$$

The position of this best matching region then determines the location of the object, ℓ*(*t*), in the current frame. Besides, $M_{c,t}^{*}$ is retained in a buffer for later updating the object appearance model *M_r_*.

### 5.3. Weakly Supervised Model Updating

As time progresses, the target object may undergo both intrinsic and extrinsic variations. Updating of the appearance model *M_r_* is thus necessary. An important issue for model updating is to ensure that the model is updated with correctly labeled samples. Contaminating the model with background samples will result in the well-known “drift” problem. Actually, tracking results collected during a certain period may contain optimal positive samples but can also have suboptimal or background samples. Previous works usually neglect this issue or simply address it by selecting good samples using a pre-fixed threshold, e.g., [19]. That is, updating is performed with samples that have distances to the object model smaller than a predefined threshold. However, during a long-term visual tracking, appearances of both the background and the target object are ever-changing. It is very difficult (if not impossible) to estimate a threshold that can separate optimal sample and suboptimal samples effectively in a long time.

To tackle this problem, our model updating method is based on two key observations that are obtained in the practice of visual tracking. First, an appearance model can effectively represent the target appearance for a certain duration. This indicates that with relatively robust appearance representation, it is not necessary to update the object appearance model too frequently. Second, in some cases there are no appropriate positive samples for updating the model. This usually happens in an occluded/absent scene where there is no “good” image region that contains the target object in that frame.

Based on the above observations, we propose to update the object appearance model by following a relatively long cycle, e.g., every 10 frames, instead of updating at each frame. Our idea is that a clustering analysis among the collected samples can naturally align similar optimal samples, suboptimal samples and background samples into different groups. The clustered group whose centroid is most close to the current object appearance model is selected for updating the object appearance model. As such, we can not only keep the appearance model adaptive to the changes but also prevent it from contamination when the tracker makes accidental tracking mistakes.

#### 5.3.1. Mean-Shift Clustering for Sample Selection

As stated in Section 5.2, the tracking result sample estimated at each frame, *i.e.*, $M_{c,t}^{*}$, is saved in a buffer that constitutes a sample set during a period of time. When the pre-fixed cycle is due, a clustering analysis is performed in the feature space among these samples. In practice, the concatenated vector representation is high dimensional. A PCA dimension-reduction procedure can be performed in advance to obtain compact representation while preserving dominant information. In fact, Ding and He proved in [53] that principal components are the continuous solutions to the discrete cluster memberships indicators for K-means clustering. It is plausible that clustering in the projected subspace may improve the clustering accuracy [53,54].

Since the tracker may occasionally make mistakes, the collected sample set can be any combination of optimal samples, suboptimal samples and background samples. It is thus very difficult to predict the number of clusters that are present. Hence, a standard clustering approach such as K-means is not appropriate. The mean shift clustering algorithm [55], which is an iterative gradient ascent method for finding local density maxima, was used instead. It does not require prior knowledge of the number of clusters and does not constrain the shape of the clusters. The data association criterion is based on the underlying probability of the data points.

**Figure 6.**
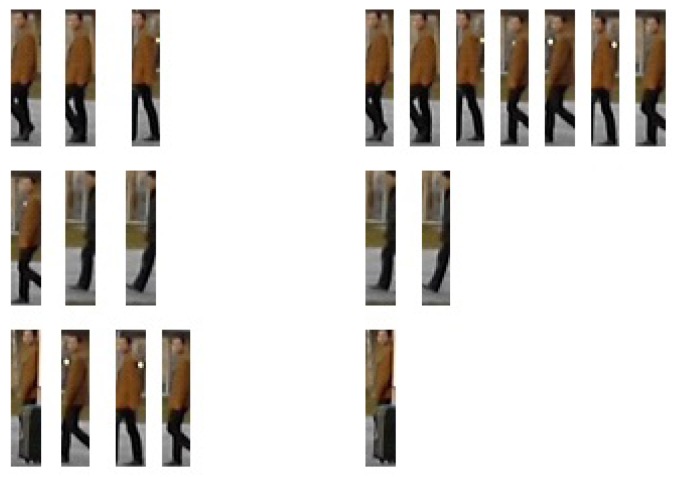
A clustering example: 10 samples on the left are simultaneously clustered into 3 groups (each row for one group) on the right according to their mutual similarities.

The algorithm begins by placing a window (actually a hyper-sphere) around each point in the feature space. In each iteration, each window moves in the direction of the mean shift vector, which is computed as follows:

(16) $$y_{t+1}=\frac{1}{\left|\Theta_{\lambda }\right|}\sum_{x\in \Theta_{\lambda }}\left(y_{t}-x\right)$$

where *y_t_* is the window center at iteration *t* and Θ_λ_ is the set of points in the hyper-sphere window of radius λ. It is also possible to use a kernel function to weight points according to how far they are from the window center. The windows eventually converge towards local density maxima yielding the cluster centroid. The points that converge to the same local maxima naturally fall into the same cluster. As such, the mean shift clustering algorithm avoids the issue of knowing the number of clusters at the price of introducing another bandwidth parameter λ. This parameter, however, is intuitive and easy to tune regarding all possible inputs [56]. An example is presented in Figure 6 to illustrate the sample clustering process.

After clustering, the arithmetic mean of each cluster is computed. Among these means, the one *M̄_s_* that has the smallest distance to *M_r_* according to Equation (14) is selected for later updating *M_r_*.

#### 5.3.2. Updating of the Object Appearance Model

As stated in Section 5.1, $M_{r}^{0}$ is preserved throughout the tracking. The updated object appearance model *M̂_r_* is determined as a linear combination of $M_{r}^{0}$, *M_r_*, and *M̄_s_, i.e.*,

(17) $$\begin{array}{l} {\hat{M}}_{r}=\alpha \cdot M_{r}^{0}+\beta \cdot M_{r}+\gamma \cdot {\bar{M}}_{s}\\ \begin{array}{ccc} s.t. & \alpha +\beta +\gamma =1.0; & 0\leq \alpha ,\beta ,\gamma \leq 1.0 \end{array} \end{array}$$

Finally, the model updating is accomplished by setting

(18) $$M_{r}={\hat{M}}_{r}$$

The advantage of employing the mixture coefficients, *i.e.*, the *α, β* and *γ*, is that they can increase the flexibility of the model. For example, with *α* set to 1.0, the model is kept fixed at $M_{r}^{0}$ and no updating is going to take place. On the other extreme, setting *γ* to 1.0 makes the model totally “forget” its appearance history.

The clustering-based appearance model updating procedure is summarized in Algorithm 2.

### 5.4. Evaluation of the Tracking System

To evaluate the performance of the proposed tracking system, we compared it with some other tracking methods on several challenging video sequences.

**Algorithm 2** Clustering-based method for updating the object appearance model.
**Input**: the most recent *N* collected samples, the initial appearance model 
$M_{r}^{0}$ the current appearance model *M_r_*.**Output:** the updated generative model *M_r_*.1:Obtain a number of clusters by performing the mean-shift clustering process in the feature space among the *N* samples.2:Compute the sample mean of each cluster.3:Find the mean *M̄_s_* that has the smallest distance to *M_r_* according to Equation (14).4:Update the object appearance model using 
$M_{r}^{0}$, *M̄_s_* and *M_r_* according to Equations (17) and (18).


#### 5.4.1. Experimental Setup

The typical settings for each component of the proposed tracking system are as follows. The feature set for the adaptive covariance descriptor is *f*(*x, y*) in Equation (12). The number of retained principal components is fixed to 14. If the whole image frame is large, the algorithm searches in a local window as specified in the dynamical model (13) to accelerate the processing speed. The size of the local search window is set to be proportional to the size of the object. Otherwise, the algorithm searches in the whole frame. In both cases, the searching step is fixed to 4 pixels horizontally or vertically.

For updating the appearance model using the clustering-based method, the updating cycle is typically set to 10–15 frames. In general, longer cycles make the model less adaptive but more stable. Besides, longer cycles can make the appearance model tolerant to longer occlusions. However, if the appearance of the object changes quickly, long updating cycle may be retarded. On the contrary, shorter cycles keep the model better up-to-date but more prone to contamination. Tradeoff between adaptivity and stability is to be considered. Setting the updating cycle to 10–15 frames can generally handle short-term tracking mistakes while keeping the model freshly adaptive to changes. In our experiments, the updating cycle was set to 10 frames. The bandwidth *h* of the mean-shift procedure is set to 1.5 for 10-dimensional vectors (after PCA dimension reduction). The linear combination coefficients *α, β* and *γ* in Equation (17) are indeed the learning the rates of the appearance model. Greater *α* is more conservative and pull the model towards the initial one $M_{r}^{0}$. Larger *γ* makes the model adapt to changes quickly but also forget its historical appearances quickly. Similar to the updating cycle, balance between stability and adaptivity is to be considered when choosing the mixture parameter for a specific application. A reference setting for *α, β* and *γ* is 0.10, 0.30 and 0.60 respectively. In our experiments, we used this reference setting for the “David-outdoor” sequence and the “White-outdoor” sequences because there are severe occlusions in these two sequences. In this case, both stability and adaptivity need to be considered. For the “Pedestrian1” sequence, we set *α, β* and *γ* to 0, 0, and 1.0 respectively, because there is no occlusion in this sequence and the appearance change is rapid.

Three benchmark sequences were used to assess the tracking performances. The first sequence is the “David-outdoor” sequence from [22], where the target undergoes partial occlusion, total occlusion, pose change and non-rigid deformation. The second one is from a self-captured video, where two pedestrians enter an outdoor campus environment, occasionally occluded by the background. This sequence contains occlusions, appearance variations and cluttered scenes. We refer to it as the “White-outdoor” sequence because the target pedestrian is in white clothes in the scene. The third sequence is the “Pedestrian1” sequence from the TLD dataset [57]. This sequence is captured by a moving camera, hence with unpredictable camera moves and large appearance variations.

We compared the proposed method with a state-of-the-art tracking method, the “MILTracker” from [58], which uses online multiple instance boosting to handle partial occlusion. Moreover, to validate the effectiveness of the clustering-based model updating method, we explicitly compared it with two other updating schemes by keeping other components the same. For noting convenience, the proposed tracking system that uses the adaptive covariance-based appearance model and the clustering-based model updating model is denoted as “AdpCov+cu”. The first comparing updating scheme is to fully adapt to the changes at every frame using the mean of current tracking result and the last model updated as in [59]. We denote the tracking system using this updating policy as “AdpCov+fu”. The other updating method is to use fixed initial model $M_{r}^{0}$ without updating. The corresponding tracking system is denoted as “AdpCov+nu”.

The sequences are labeled with the “ground truth” for each frame. Percentage of Correctly tracked Frames (PCF) was employed to quantitatively measure the performance of all the involved trackers. PCF computes the percentage of correctly tracked frames over the total number of frames in the sequence. Tracking is considered to be correct if the overlap of the bounding box of the tracking result and that of the ground truth is greater than 25% of the area of the ground truth.

#### 5.4.2. Results

**Figure 7.**
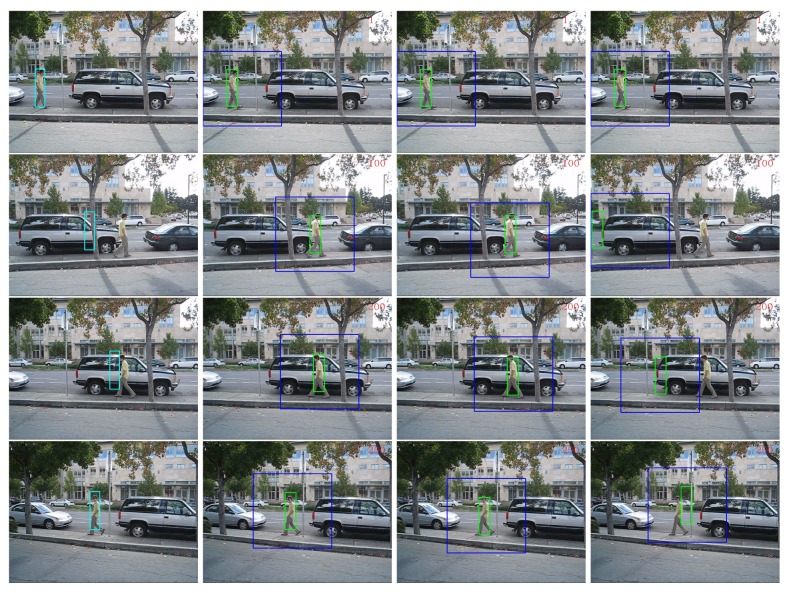
Tracking results on the “David-outdoor” sequence using different updating policies. Column 1: “MILTracker”. Column 2: “AdpCov+cu”. Column 3: “AdpCov+nu”. Column 4: “AdpCov+fu”. In the last three columns, the blue rectangles indicate the search windows and the green rectangles show the tracking results. Row 1: Frame #1. Row 2: Frame #100. Row 3: Frame #200. Row 4: Frame #250.

**Figure 8.**
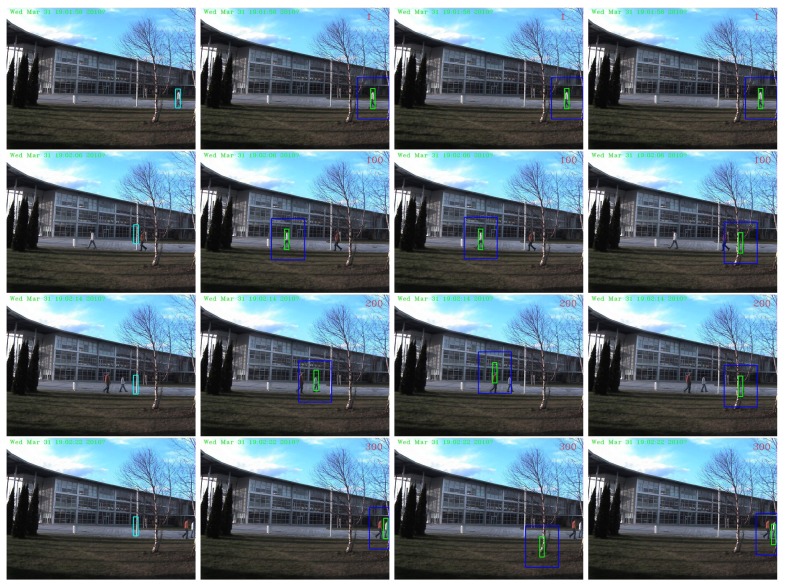
Tracking results on the “White-outdoor” sequence using different update policies. Column 1: “MILTracker”. Column 2: “AdpCov+cu”. Column 3: “AdpCov+nu”. Column 4: “AdpCov+fu”. In the last three columns, the blue rectangles indicate the search windows and the green rectangles are the tracking results. Row 1: Frame #1. Row 2: Frame #100. Row 3: Frame #200. Row 4: Frame #300.

**Figure 9.**
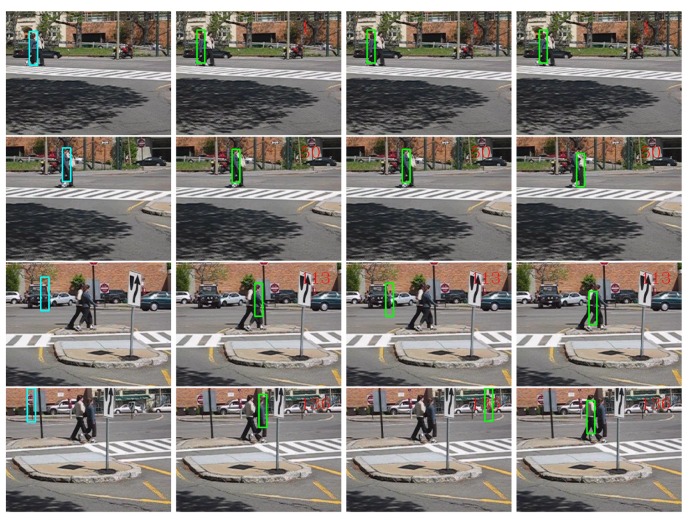
Tracking results on the “Pedestrian1” sequence using different update policies. Column 1: “MILTracker”. Column 2: “AdpCov+cu”. Column 3: “AdpCov+nu”. Column 4: “AdpCov+fu”. Row 1: Frame #1. Row 2: Frame #50. Row 3: Frame #113. Row 4: Frame #136.

The performances in terms of PCF of all the comparing tracking methods are presented in Table 2. The “AdpCov+cu” achieved the best results on all the three sequences. For qualitative comparison, we display a few screen snapshots of tracking results on the “David-outdoor” sequence, the “White-outdoor” sequence and the “Pedestrian1” sequence using all the comparing tracking methods in Figures 7–9 respectively [60–62].

The “MILTracker” generally fails when there are severe occlusions (see Frame #100 in Figure 7) or fast appearance changes (see Frame #113 in Figure 9). The “AdpCov+nu” tracker drifts to non-targets when the target undergoes significant appearance deformation (see Frame 200 and Frame 300 in Figure 8) or when there are similar non-targets in the scene (see Frame #113 in Figure 9). On the other hand, the “AdpCov+fu” tracker usually leads to tracking failure because its object appearance model is eventually contaminated due to updating during occlusion (see Frame #100 in Figure 8) or accumulated tracking errors (see Frames #50, #113 and #136 in Figure 9).

We see from the above results that the clustering-based updating method can effectively tolerate short-term tracking mistakes, including drifting to non-targets, partial/full occlusions, keeping the object appearance model steadily attached to the object and up-to-date. The integrated tracking system “AdpCov+cu” accomplished stable tracking and outperformed a state-of-the-art tracker.

## 6. Conclusions

We have analyzed the generalization ability of the covariance descriptor and revealed that small eigenvalues may incur large variance and thus degrade its generalization. Generally, fusing more features can yield better detection performance, as long as it does not incur the small eigenvalue problem. When there are very small eigenvalues, the covariance descriptor hardly generalize. It is the logarithm function in the distance metrics that causes large disturbance and thus degrades the descriptor's generalization ability.

Regularization can effectively cure this problem and meanwhile preserve all the information in the corresponding dimensions. PCA dimension reduction, on the other hand, removes the unreliable dimensions, which can also be viewed as an adaptive feature extraction process. As dimensionality is reduced, operations on the adaptive covariance descriptor are generally less time-consuming.

The clustering-based updating method is able to select a group of reliable samples to update the object appearance model, which is particularly useful when there are tracking mistakes during tracking. Compared with other updating schemes, the clustering-based method showed merits in both adaptivity and stability.

The tracking system that integrates the adaptive covariance descriptor and the clustering-based updating has accomplished stable tracking in several challenging real-world video sequences and outperformed a state-of-the-art tracker. We thus believe that the two components proposed in this work can serve as building blocks for constructing more robust tracking systems.

## Figures and Tables

**Table 1.** Detection performance using the conventional covariance descriptor, the regularized covariance descriptor and the adaptive covariance descriptor. Frames where the object is severely occluded are not counted in the performance computation.

**Sequence**	**Detection Rate**						**Time Per Frame in Seconds**		
	
	**Cov1**	**Cov2**	**RegCov1**	**RegCov2**	**AdpCov1**	**AdpCov2**	**Cov**	**RegCov**	**AdpCov**
PkTest01	84%	83%	100%	91%	100%	91%	1.614665	1.50845	0.42641
PkTest02	69%	69%	91%	75%	82%	68%	1.798325	1.173205	0.48842
PkTest03	100%	78%	100%	88%	100%	90%	2.199425	2.005465	1.043075
PETS	100%	98%	100%	100%	100%	98%	4.02714	2.88185	2.952495

**Table 2.** Information of the sequences and the tracking performances in terms of PCF.

**Sequence**	**David-Outdoor**	**White-Outdoor**	**Pedestrian1**
Number of frames	251	305	140
Frame size	640 × 480	640 × 480	320 × 240
Initial object size	38 × 126	16 × 70	16 × 65
Severe occlusion(s)	Twice	4 times	None

MILTracker	48.4%	9.84%	69.78%
AdpCov+cu	96.0%	93.44%	97.84%
AdpCov+nu	96.0%	68.20%	61.15%
AdpCov+fu	12.8%	6.89%	28.78%
